# The use of rapid and cost-effective blood-based biomarkers in combination with tumour TNM stage for individual head and neck cancer patient treatment selection

**DOI:** 10.1007/s12032-017-0912-7

**Published:** 2017-03-18

**Authors:** Nongnit Laytragoon Lewin, Freddi Lewin, Bengt-Åke Andersson, Sture Löfgren, Lars Erik Rutqvist

**Affiliations:** 1 Division of Medical Diagnostic, Ryhov Hospital, 55322 Jönköping, Sweden; 2 Department of Clinical and Experimental Medicine, Linköping University, 58185 Linköping, Sweden; 3 Department of Oncology, Ryhov Hospital, 55322 Jönköping, Sweden; 4 Swedish Match AB, 11885 Stockholm, Sweden

**Keywords:** Head and neck cancer, Single-nucleotide polymorphism, Inflammation, Individualized treatment selection

## Abstract

Head and neck (H&N) cancer is an aggressive disease and the incidence has increased in younger population worldwide. Tumour TNM staging is the main basis for treatment decision despite significant variation in clinical outcome. Survival time of these patients has marginally improved during the last 30 years. Various biomarkers with cumbersome analysis, high cost, time consumption and requirement of special laboratory facilities have been investigated. However, none of these biomarkers have been shown to be suitable to use for individual H&N cancer patient treatment selection in the clinic. For practical use in clinical settings, the given biomarkers must be simple to analyse, rapid, cost effective and available in routine laboratories. With this intension, we suggested the combination of standard TNM staging and biomarkers associated with inflammation such as neutrophils, neutrophil to lymphocyte ratio, plasma C-reactive protein or plasma tumour necrosis factor alpha (TNFa) and single-nucleotide polymorphism in TNFa rs1800629 using blood-based analysis. The optimal treatment outcome of H&N cancer by using combination of TNM stage and these blood-based biomarkers for individual patient selection need further investigation.

## Introduction

Head and neck (H&N) cancer is an aggressive disease and the incidence is increasing, especially in younger population worldwide [1, 2]. Relatively high loco-regional recurrent tumours, derived from local primary tumour or second primary tumour from the normal-appearing tissue were observed in these patients [3–5]. Despite aggressive and advanced treatment regimens, long-term survival in human papilloma virus (HPV) negative H&N cancer is only marginally improved during the last 30 years [2, 6–12].

Tumour TNM staging is the primary guide for treatment modality despite significant heterogeneity in clinical outcome. Independent from TNM stage, number of circulating tumour cells, lymphocytes expressing cytolyses granules, neutrophils, neutrophil to lymphocyte ratio (NLR), plasma C-reactive protein (CRP), tumour necrosis factor alpha (TNFa) or downregulated T cell receptor Zeta chain was associated with the clinical outcome of cancer patients [13–19].

Prognostic biomarkers for individual patient treatment selection in the clinical practice need to be established for optimal clinical outcome of H&N cancer patients. We emphasize the cost-effective, rapid and automatic blood-based biomarkers in combination with TNM staging for individual treatment selection in the clinical setting of this patient group.

## HPV, cigarette, alcohol, single-nucleotide polymorphism (SNP) and H&N cancer

Epidemiological data have established that cigarette smoking, alcohol consumption or HPV infection are the main risk factors for H&N cancer [20–23]. More than 95% of these malignant tumours are squamous cell carcinoma, originating from the mucosal epithelium cells linings of the upper aero-digestive tract [24]. It is important to point out that not all HPV infected persons, heavy smokers or high alcohol consumers develop cancer. The elevated cancer risk among first-degree relatives of H&N cancer patients indicated an important role of genetic factors [25, 26].

SNP is the most common source of human genetic variation in the individual germline DNA. SNP sequence variation may lead to altered effects of the gene and causes inter-individual susceptibility to oncogenesis. One SNP, TNFa rs1800629 is associated with increased risk of H&N cancer, tumour recurrence and survival time of the patients [27].

HPV is a common sexually transmitted infection and most of the sexually active women and men worldwide are infected by low-risk or high-risk HPV in their life time [22, 28, 29]. HPV infect squamous epithelial cells through cellular membrane receptors [29–31]. Sexual behaviour, oral hygiene and smoking are suggested to be related to oral HPV infection in the healthy individuals [32, 33].

High-risk HPV type might be necessary for tumorigenesis but it is not sufficient on its own [22]. Individual host SNPs influences the susceptibilities to high-risk or low-risk HPV infection process and outcome of the infection [33]. Generally, virus infected cells will carry virus genome in the episomal form, maintained at constant copy numbers and replicated along with host cellular DNA [29, 34]. Host immune response plays a role in clearance of HPV since HPV infected epithelial cells are spontaneously regressed in immunocompetent individuals [22, 35, 36].

The presence of high-risk HPV-DNA is insufficient to classify accurately tumours as an HPV associated since it might be present as the passenger, inactive virus and not the cause of malignancy [37, 38]. The oncogenesis of high-risk HPV is suggested to occur from integrated high-risk HPV-DNA in the host DNA [36]. This high-risk, integrated HPV-DNA acquires the capacity to inhibit cellular differentiation and actively transcribes high-risk HPV oncoproteins in host cells [39, 40].

Two techniques, PCR and in situ hybridization (ISH), are generally used for diagnosis of high-risk HPV status in biopsy material. The presence of high-risk HPV-DNA shown by PCR or ISH is, however, insufficient to accurately classify tumours as HPV associated. Fluorescence in situ hybridization (FISH) could identify whether the high-risk HPV is oncogenic with integrated HPV-DNA or inactive with episomal HPV-DNA in the infected cells [40]. This analysis is, however, not suitable in clinical practice due to time consumption, high cost and requirement of special laboratory facilities.

Cigarette smoking and alcohol consumption are independent risk factors but when combined a synergistic effects is observed [20]. In vitro, combination of smoke extract and alcohol induces massive normal cell death and this is associated with SNP genotype of the individual donors [41, 42]. High frequency of HPV infection in multiple dysplastic lesions of smokers or alcohol users were detected [43–46]. An additive impact of SNPs, HPV, smoking and alcohol consumption on risk to develop H&N cancer could be expected [45–48].

## Inflammation, SNPs and clinical outcome of H&N cancer patients

Systemic inflammation from smoking induces massive cell death in the body might indicate by increasing circulating neutrophil, NLR, perforin expression in T cells and plasma CRP or TNFa levels were detected in healthy smokers (Luetragoon et al., manuscript in preparation). HPV infection and alcohol consumption could also increase normal cell death [22, 41, 42, 49]. Independent from etiological point of view, cell death will release autologous antigens that provoke local and systemic host immune response [33]. Long-term exposures to these autologous antigens will upregulated inflammatory biomarkers, and this seems to be correlated with specific SNP sequence and not random events [26, 47].

Human solid tumours are generally infiltrated by inflammatory cells and considered to be a host attempt at the detection of emerging tumour cells and their elimination [50]. H&N tumours are among the most highly immune-infiltrated cancer types. The smoking patient with tumours harbouring mutation signatures and lower immune infiltration had a shorter survival time [51, 52].

Elevated frequency and enhanced suppression capacity of regulatory T cells (Treg) was detected in blood circulation of H&N cancer patients [53, 54]. Increased levels of circulating plasma CRP and TNFa was associated with survival of H&N cancer patient [15–17, 27]. Thus, tumour and the inflammatory cells might acquire the immunosuppressive activity or secretion inflammatory cytokines [55, 56].

## Blood-based genetic and inflammation biomarkers in H&N cancer patients

Peripheral blood contains immune response cells, plasma proteins, cytokines and variable mediators with wide-ranging biologic effects. Circulating blood are in direct contact with all organs, non-invasive to obtain and suitable for transportation. Analysis of SNPs and inflammatory biomarkers could be simply done using this peripheral blood sample.

Using only TNM stage as treatment selection might be practical but not optimal to predict clinical outcome of H&N cancer patients. Independent from tumour etiological or TNM stage, SNP in TNFa and elevating number of inflammatory biomarkers obtained from pre-treatment peripheral blood sample is associated with the clinical outcome of H&N cancer patients [7, 15–17, 27]. Analysis of SNPs and these inflammation biomarkers using peripheral blood samples are well established. They are rapid, cost effective and can be done with automatic equipment, existing in the routine laboratory.

With intension to be used as biomarkers in clinical practice, such analysis must be rapid, available in routine laboratory facility and cost effective. For optimal personalized H&N cancer treatment, we propose the pre-treatment blood-based biomarkers with focus on TNFa rs1800629, neutrophil counts, NLR, plasma CRP and TNFa in combination with standard pathological diagnosis and TNM classification for individual H&N cancer patient treatment selection. The prognostic value of this combination warrants future clinical investigation.

## Conclusion

The impact of genetic variation, smoking, alcohol consumption, HPV infection and HPV integration significantly influence host immune response [22, 23, 31, 42, 52]. This could facilitate the formation of an inflammation microenvironment and systemic inflammation that favours carcinogenesis and cancer progression in H&N cancer patients [15, 17, 23, 27]. The clinical significance of SNPs and systemic inflammatory biomarkers investigated at the diagnostic time, suggests the possibility of individual patient treatment selection [7, 15, 16, 18, 27].

The analysis of TNFa rs1800629 and systemic inflammatory biomarkers such as plasma CRP, TNFa or neutrophil and NLR using blood sample are rapid, cost effective and require only common facility in the routine laboratory. Thus, blood-based biomarkers offer an attractive possibility to use in combination with standard tumour TNM stage for individual H&N cancer patient treatment selection in the clinical setting. The prognostic value of TNM stage combined with these blood-based biomarkers for treatment selection of H&N cancer patients warrants further investigation.
